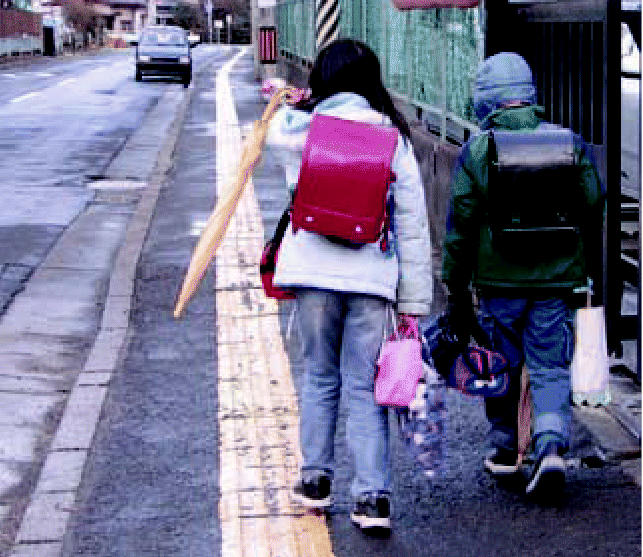# Meeting Report: Looking Hard at Early Exposures

**Published:** 2006-10

**Authors:** Kimberly Thigpen Tart

Children’s health was the focus of almost 30 different sessions at the International Conference on Environmental Epidemiology and Exposure, held 2–6 September 2006 in Paris. Exposure to environmental toxicants early in life, and even parental exposure prior to conception, may lead to metabolic effects, cardiovascular disease, and reproductive problems later in life, said researchers speaking at the meeting.

According to Germaine Buck Louis, an epidemiologist from the National Institute of Child Health and Human Development, there is scientific evidence to support a relationship between maternal and paternal exposures prior to conception and testicular dysgenesis syndrome, a collection of adverse effects in testes, as well as the less well-studied ovarian dysgenesis. Said Buck Louis, “Periconception is a vital stage for research on exposures.”

George Davey-Smith, a researcher in the University of Bristol Department of Social Medicine, told plenary attendees that prenatal and early-life exposures to environmental factors such as infectious agents and tobacco smoke have been associated with effects on blood pressure, insulin resistance (possibly leading to obesity), and cardiovascular disease in adults. Smith says that so-called predictive adaptive responses—developmental “programming” in response to adverse environmental cues—may underlie some of these associations. Evolutionarily, these responses are intended prepare the developing organism for a life of hardship. For example, a mother’s poor nutrition during pregnancy may “predict” a life of nutritional hardship for her fetus. The resulting changes in fetal metabolic and cardiovascular development can prove maladaptive if the child is not, in fact, nutritionally deprived; the result can be metabolic syndrome.

In a particularly packed session on children’s health and environmental chemicals, Brenda Eskenazi, a professor of epidemiology at the University of California, Berkeley, presented the as yet unpublished, compiled results of studies from three NIEHS/EPA Children’s Environmental Health Centers (UC-Berkeley, Columbia University, and Mount Sinai Medical Center) that showed similar associations between exposure to organophosphate pesticides and neurodevelopmental effects. Said Eskenazi, “The children’s centers have begun to come of age, and we can now reap the benefits of harmonizing the methods for our longitudinal birth cohort studies. It demonstrates the importance of conducting similar studies in different populations.”

Promotion of research on how adverse environmental exposures affect children is the primary goal of a new society announced at the Paris meeting. The International Society for Children’s Health and the Environment is dedicated to protecting children from adverse environmental influences—chemical, physical, biological, and social—through research, training, policy making, clinical care, and education. The society also seeks to promote children’s health by enhancing the quality of their environments.

Bruce Lanphear, director of the Children’s Environmental Health Center at Cincinnati Children’s Hospital Medical Center, is a founding member of the new society. He said, “It has become increasingly clear that children are particularly vulnerable to numerous environmental hazards. This society is being formed to combine and enhance our efforts to protect children from these hazards.” The society is currently recruiting members.

Protection of children’s health is part of the basis for a joint declaration by scientists of the International Society for Environmental Epidemiology and the European Respiratory Society, presented at the meeting, which calls for European Union authorities to strengthen proposed air quality directives. Bert Brunekreef, a professor of environmental epidemiology at the University of Utrecht, said, “It is now widely recognized that the [current] levels are not completely or sufficiently protective of public health.” He added that the proposals “lack teeth” because they do not include mandatory requirements for reduction of fine particle pollution, which is associated with increases in asthma attacks and decreased pulmonary function in children. The European Commission is expected to review the proposed directives on 13 October 2006.

## Figures and Tables

**Figure:**